# Autosegmentation of the rectum on megavoltage image guidance scans

**DOI:** 10.1088/2057-1976/aaf1db

**Published:** 2019-01-10

**Authors:** L E A Shelley, M P F Sutcliffe, K Harrison, J E Scaife, M A Parker, M Romanchikova, S J Thomas, R Jena, N G Burnet

**Affiliations:** 1 University of Cambridge, Department of Engineering, Cambridge, United Kingdom; 2 Addenbrooke’s Hospital, Department of Medical Physics and Clinical Engineering, Cambridge, United Kingdom; 3 Cambridge University Hospitals NHS Foundation Trust, Cancer Research UK VoxTox Research Group, Cambridge, United Kingdom; 4 University of Cambridge, Cavendish Laboratory, Cambridge, United Kingdom; 5 Gloucestershire Oncology Centre, Cheltenham General Hospital, Cheltenham, United Kingdom; 6 National Physical Laboratory, Teddington, United Kingdom; 7 Addenbrooke’s Hospital, Oncology Centre, Cambridge, United Kingdom; 8 University of Manchester, Manchester Academic Health Science Centre, Manchester, United Kingdom; bpexa1dbem1 ls698@cam.ac.uk

**Keywords:** autosegmentation, adaptive radiotherapy, image guidance, delivered dose, rectal contouring, deformable image registration

## Abstract

Autosegmentation of image guidance (IG) scans is crucial for streamlining and optimising delivered dose calculation in radiotherapy. By accounting for interfraction motion, daily delivered dose can be accumulated and incorporated into automated systems for adaptive radiotherapy. Autosegmentation of IG scans is challenging due to poorer image quality than typical planning kilovoltage computed tomography (kVCT) systems, and the resulting reduction of soft tissue contrast in regions such as the pelvis makes organ boundaries less distinguishable. Current autosegmentation solutions generally involve propagation of planning contours to the IG scan by deformable image registration (DIR). Here, we present a novel approach for primary autosegmentation of the rectum on megavoltage IG scans acquired during prostate radiotherapy, based on the Chan-Vese algorithm. Pre-processing steps such as Hounsfield unit/intensity scaling, identifying search regions, dealing with air, and handling the prostate, are detailed. Post-processing features include identification of implausible contours (nominally those affected by muscle or air), 3D self-checking, smoothing, and interpolation. In cases where the algorithm struggles, the best estimate on a given slice may revert to the propagated kVCT rectal contour. Algorithm parameters were optimised systematically for a training cohort of 26 scans, and tested on a validation cohort of 30 scans, from 10 patients. Manual intervention was not required. Comparing Chan-Vese autocontours with contours manually segmented by an experienced clinical oncologist achieved a mean Dice Similarity Coefficient of 0.78 (SE < 0.011). This was comparable with DIR methods for kVCT and CBCT published in the literature. The autosegmentation system was developed within the VoxTox Research Programme for accumulation of delivered dose to the rectum in prostate radiotherapy, but may have applicability to further anatomical sites and imaging modalities.

## 1. Introduction

Automated segmentation of the anatomy, or autosegmentation, is crucial for optimising the efficacy of adaptive radiotherapy (ART) (Jaffray *et al* 2010, Godley *et al* 2013, Thor *et al* 2013, Whitfield *et al* 2013). Reactive adaptations to a patient’s radiation treatment plan may be necessary if anatomical changes occur during treatment resulting in deviations from the intended planned dose. Image guided radiotherapy (IGRT) facilitates visualisation of the patient’s anatomy throughout the course of treatment and offers a potential platform for assessing dosimetric implications. However, the expanse of information contained within IGRT images is not currently being realised to its full potential, and this is partly due to the dependency on manual contouring.

The development of robust and automated approaches to segmentation has been identified as a key aspect in the pursuit of delivered dose calculation for ART (Jaffray *et al* 2010), as manual contouring of daily IG scans is unfeasible. Not only would this introduce an impracticable excess to the clinical workload (Gambacorta *et al* 2013, Scaife *et al* 2014), but additional training would be required due to the poorer soft tissue definition of IG scans when compared with the more familiar kilovoltage (kV) treatment planning scans (Whitfield *et al* 2013). The reduction in image quality is due to the lower contrast and signal-to-noise ratio associated with cone-beam computed tomography (CBCT) and megavoltage CT (MVCT) imaging (Chao *et al* 2008, Jackowiak *et al* 2015). Automated solutions present the opportunity to expedite and standardise anatomical segmentation of IG scans (Weiss *et al* 2010, Gambacorta *et al* 2012, Gambacorta *et al* 2013). Approaches for autosegmentation to date have generally focused on intensity values, atlas-based tools, or shape-based models, each with their own limitations (Whitfield *et al* 2013).

The purpose of this work is to develop an autosegmentation tool to identify the rectum on MVCT IG scans for patients undergoing prostate IGRT. This review of the literature focusses on segmentation tools relevant to this anatomy. The motivation is that daily segmentation could facilitate quantitative tracking of interfraction rectal motion and deformation throughout the course of treatment (Scaife *et al* 2014). Deviations in rectal positioning from the planning CT scan have been shown to induce differences between the intended planned dose, and that actually received (Scaife *et al* 2015, Shelley *et al* 2017). However, segmentation of anatomy within the pelvic region can be particularly challenging. Soft tissue boundaries lack distinction and worsen in low contrast imaging (Lütgendorf-Caucig *et al* 2011, Geraghty *et al* 2013). Methods used for segmentation of the prostate, such as deformable image registration (DIR), are generally not applicable for the rectum due to the large and unpredictable spatial deformations caused by rectal contents and intestinal gas (Michalski *et al* 2010, Niu *et al* 2012, Scaife *et al* 2014, Varadhan *et al* 2015). Common DIR algorithms struggle due to intensity variations and the lack of one-to-one correspondence between a full, gassy, or empty rectum (Chao *et al* 2008, Niu *et al* 2012, Zambrano *et al* 2013). Previous studies investigating the dosimetric effects of interfraction rectal motion have been dependent upon manual delineation of the rectum on IG scans (Kupelian *et al* 2006, Sripadam *et al* 2009, Chen *et al* 2010, Anderson *et al* 2011, Hatton *et al* 2011, Peng *et al* 2011, Mcparland *et al* 2014, Pearson *et al* 2016, Collery and Forde 2017), consequently being limited in sample size. One approach attempting to address this limitation was to implement statistical simulations for quantifying motion-inclusive delivered dose (Thor *et al* 2013). A common recommendation of these studies was the development of robust systems for autosegmentation of the rectum, as a crucial component towards achieving automated ART for prostate radiotherapy.

Autosegmentation of rectal contours has previously been addressed for standard kVCT imaging. Evaluations of selected commercial algorithms by Geraghty *et al* 2013, and La Macchia *et al* 2012, found that systems struggled to identify the rectum on the planning kVCT without manual intervention. Despite the superior image quality of kVCT, the prostate-rectum interface was affected by poor or no contrast (particularly at the superior and inferior rectal boundaries) which led to greater inter-observer error. It follows that these difficulties would worsen for poorer quality IG scans. However, Zambrano *et al* 2013, found no significant differences in rectum registration errors between kVCT-kVCT and CBCT-kVCT using an in-house featurelet-based model, though concluded that their DIR accuracy was not yet sufficient for clinical contour propagation. Gao *et al* 2006, proposed an intensity modification method (IMM) based on an in-room diagnostic kVCT-on-rails system, which introduced artificial gas with adaptive smoothing. The IMM improved upon rigid transformation and DIR alone. However, standard kVCT imaging is not often available for IG, and in our study we sought to exploit images already routinely acquired during treatment.

Autosegmentation techniques developed for standard kVCT may not be transferrable to lower-quality IG scans (Whitfield *et al* 2013). Alternative approaches have been proposed for CBCT, the most common IG system since being fitted as standard to modern gantry-based linear accelerators. Commercial systems are beginning to support DIR of IG scans (Brock *et al* 2017), including the implementation of advanced hybrid methods rather than intensity-based approaches (Takayama *et al* 2017). Several research groups have investigated independent solutions for autosegmentation of the rectum. Chao *et al* 2008, describe a narrow shell warping technique to map the rectal contour via b-spline DIR from planning kVCT to CBCT, achieving a mean error of 2 mm. This complemented the methods of Xie *et al* 2008, who applied scale invariance feature transformation and thin plate spline transformation to a set of control points surrounding the rectum, resulting in over 90% accordance between manually segmented and DIR mapped rectum. Chen *et al* 2009 reported similar results using a modified Demons algorithm based on CBCT greyscale. Thor *et al* (2011, 2013) found the modified DIR Demons algorithm system struggled with large rectal deformations, resulting in only 20% of propagated rectal contours being classified as good or acceptable. As such, translation of these tools into fully automated ART has not yet been achieved in clinical practice.

DIR has been successfully applied to MVCT imaging in the context of phantom measurement and patients with head & neck cancer (Nobnop *et al* 2017, Yeap *et al* 2017), but as described for kVCT and CBCT, is less accurate when boundaries are poorly defined. MVCT image quality is adequate for visualising the rectum (Yang *et al* 2009) and bowel (Perna *et al* 2016), but successful DIR contour propagation has been dependent upon manual contouring or intervention. Studies reporting on DIR of MVCT (Kupelian *et al* 2006, Wahl *et al* 2017), or MV-CBCT (Akino *et al* 2013) for dose accumulation of the rectum have relied upon manual segmentation, and as such have been limited in terms of patient numbers. For identifying the rectum on MVCT scans, alternatives to DIR such as independent primary segmentation approaches, may be more appropriate in the context of achieving dose accumulation for ART.

Here we present a novel method that has been developed to automatically identify the rectum on daily MVCT scans acquired for patients undergoing IGRT to the prostate using TomoTherapy® (Accuray, Sunnyvale, CA). The basis of the contouring is the Chan-Vese algorithm (Chan and Vese 2001), implemented in 2D within the MATLAB coding environment (MathWorks®, Natick, MA). As such, the difficulties previously described for using DIR to identify the rectum are avoided. Full details are provided, including the use of prior knowledge, rigid registration for setup correction, image windowing, and identification of poor contours. The algorithm was developed on training data, and validated on test scans, before integration into the VoxTox research programme (Burnet *et al* 2017). We demonstrate that IG scans have further use than routine positional verification by extracting quantitative information in the form of anatomical contours from these images. No additional exposures were required to obtain the contours, as IG was already included in the patient pathway. Contrary to the methods discussed above, our approach performs primary segmentation rather than contour propagation, which addresses the challenges associated with the magnitude of shape change and intensity variation observed in the rectum.

## 2. Material and methods

### 2.1. Clinical imaging details

The VoxTox research programme is an observational study investigating the link between delivered radiation dose and toxicity (Burnet *et al* 2017). All patients were treated with TomoTherapy® (Accuray, Sunnyvale CA), with daily MVCT image guidance scans acquired immediately prior to treatment for the purposes of online positional verification. The VoxTox study received approval from the National Research Ethics Service (NRES) Committee East of England (13/EE/0008) in February 2013 and is part of the UK Clinical Research Network Study Portfolio (UK CRN ID 13716).

An experienced clinician [JES] manually delineated the rectum on 56 MVCT IG scans from 10 prostate cancer patients (approximately 560 slices). These contours were taken as the gold standard when evaluating the accuracy of the autosegmentation. On a subset of 6 scans from the same patients, the rectum was independently delineated by 8 oncologists, including JES (Scaife 2015, Burnet *et al* 2017). The median Jaccard Conformity Index, JCI (Jaccard 1901), of JES relative to the other observers was 0.83, giving a measure of the inter-observer variability. Twenty-six scans were used to train the autosegmentation algorithm, and 30 test scans were used for validation of the generated autocontours. Test scans were distinct from training scans, and autocontours were visually reviewed by JES.

Imaging specifications for the kVCT were: 272 × 272 pixels per slice, pixel size 1.953 mm, slice thickness 3 mm. Scan length included the full extent of the rectum, from rectosigmoid junction to the most inferior slice containing both ischial tuberosities (Scaife *et al* 2014). MVCT specifications were: 512 × 512 pixels per slice, pixel size 0.754 mm, slice thickness 6 mm. The field of view for MVCT imaging was limited to typically 8–12 slices according to local protocols to minimise additional dose and time for prostate IGRT (Bates *et al* 2013), so only a proportion of the rectum was imaged.

### 2.2. Algorithm overview

**Figure 1.**
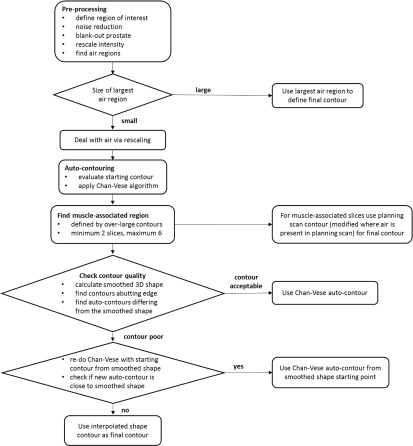
Flow diagram describing autosegmentation algorithm.

Figure 1 shows a flow diagram summary of the algorithm for rectal contour detection. The best estimate of the rectal contour is taken from either: (i) a region with air, (ii) the kVCT planning contour for the muscle-associated region (either as-is or modified where air is present in the planning scan), (iii) the autosegmentation result (either from the initial pass or using the smoothed shape for a revised starting contour), or (iv) an interpolated contour. All steps are described in the following sections. It is important to note that identification of the best choice of contour is intrinsic to the algorithm, and does not require manual intervention.

### 2.3. Pre-processing

The following pre-processing steps are applied to MVCT scans to optimise autosegmentation of the rectum. First, a rigid registration is performed to align the daily image with the kVCT scan. The shifts and rotations of this registration replicate the couch positional adjustments applied by the treatment radiographers on set, and are obtained from TomoTherapy archives (Romanchikova *et al* 2018). Once registered, a median filter of width 5 pixels is applied to reduce noise, image intensities are rescaled to improve contrast, and any arising complexities due to air pockets are addressed. These steps are detailed below.

#### 2.3.1. Rescaling hounsfield units

**Figure 2.**
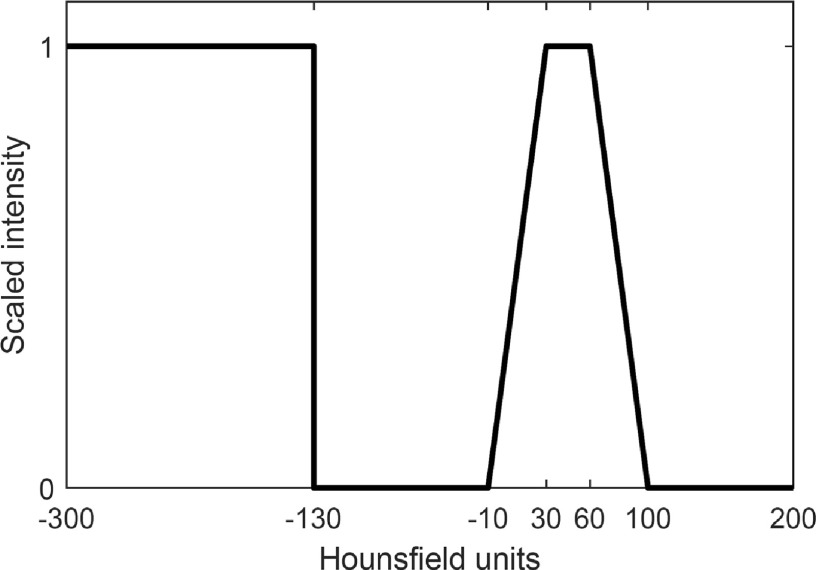
Rescaling from Hounsfield Units (HU) to scaled intensity. Pixels less than −130 HU are identified as rectal gas and are rescaled to an intensity value of 1 to be included as rectal content. Pixels between −10 HU and 100 HU are in the ‘critical range’ identified as rectal contents, and are rescaled to intensity values between 0 and 1.

To enhance contrast between tissue, air, and bone, the MVCT Hounsfield Units (HU) are re-scaled to intensity values between 0 and 1, as illustrated in figure 2. Rescaling parameters were selected based on clinically optimal windowing parameters. The contrast between the rectum and surrounding material is improved by assigning the rectal wall and contents an intensity approaching 1, and surrounding tissue an intensity approaching 0. In the ‘critical range’ found for rectal contents between −10 HU and 100 HU (derived from a set of examined scans), pixels are rescaled and assigned an intensity value between 0 and 1. Pixels between 30 HU and 60 HU are assigned an intensity of 1, with linear ramps up to these values as shown in figure 2. The linear ramp function is a simple method for applying extra weighting to material identified as lying within the rectum, ramping off as it becomes less certain whether material should be included in the rectum. Pixel values greater than 100 HU are assigned an intensity of 0. Pixels values less than −130 HU are assumed to be gas pockets within the rectal contour so are assigned an intensity value similar to rectal matter to aid the autosegmentation process. The rectal gas threshold of −130 HU was determined empirically and differs from the standard air value of −1000 HU due to traces of solid/liquid matter in the air pockets, and partial volume effects. Larger gaseous regions are treated as a special case and are discussed below.

#### 2.3.2. Search region

To increase the robustness and efficiency of the algorithm, a search region is defined on the MVCT image by expanding around the original location of the rectum, identified using prior knowledge of the kVCT planning scan rectal contour following rigid registration. The area of expansion of the region of interest (ROI) is based on the rectum’s maximum estimated displacements, obtained from a consideration of rectal contours defined manually by several clinicians (Scaife *et al* 2014). Values of the expansion on the MVCT scan are taken as 38 mm (50 pixels) anteriorly, 15 mm (20 pixels) posteriorly, and 30 mm (40 pixels) left and right. In addition, *a posterior* limit of the ROI is defined by the location of the spine, if present and identifiable on a given slice (using a thresholding approach).

#### 2.3.3. Dealing with air

**Figure 3.**
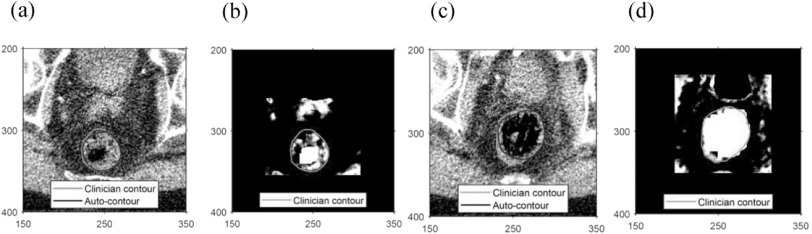
Image HU/intensity rescaling applied to regions containing air regions: (a) original and (b) rescaled scans with a small air region; (c) original and (d) rescaled scans with a larger air region. Portions of the scan outside the region of interest are black in the rescaled scans. In both cases, the spine at the bottom of the scan has been detected and used to identify the most posterior margin of the region of interest. Note that, in (d), the prostate lying anterior to the rectum has been rescaled to dark pixels. Axes are labelled in pixels.

The presence of air, or rectal gas, in the scan provides a useful marker of the rectum, but is also a potential source of confusion to an automated algorithm. For each MVCT image slice, the largest region of connected air pixels, as determined following intensity scaling, is found using the MATLAB *regionprops* function. For regions of air spanning approximately 85 to 340 mm^2^ (an area of 150 to 600 pixels), the largest connected region is identified, with any resulting ‘holes’ filled in. The region is enlarged by 6 mm (8 pixels) to allow for surrounding rectal wall. Intensity re-scaling serves to ensure that smaller gas regions less than 85 mm^2^ in area tend to be included within the rectal contour on applying the autosegmentation algorithm. Regions identified as rectal gas spanning over 340 mm^2^ are explicitly included within the rectal contour by simply defining the rectal contour as this area plus a margin to account for the rectal wall. In addition, some smoothing of the contour is applied to give a realistic solution. Figure 3 illustrates two cases for dealing with smaller (a and b), and larger (c and d) air regions. Autocontours derived from the air regions are shown in the original scans, figures 3(a) and (c), and are shown alongside the clinician-defined contours for comparison.

Some spuriously detected ‘air regions’ are disregarded if the centre of the air region does not lie within the location of the original rectal planning scan contour.

In some cases, the kVCT planning contours are propagated to determine the best estimate of a particular MVCT slice, as detailed below. To account for potential changes in the MVCT rectal contour due to air, the area of air in the kVCT scan is evaluated using the above approach. The kVCT rectal contour is then reduced (using the MATLAB erosion function) by the difference between the kVCT- and MVCT-determined air areas, to produce the best estimate for the MVCT rectal contour for that slice.

#### 2.3.4. Dealing with the prostate

Because the MVCT IG scans are used for target localisation during treatment, the assumption is made that the location of the prostate is consistently positioned between scans. Since the prostate and rectum do not overlap, pixels on the MVCT scan included within the original kVCT prostate contour are avoided by the rectal autosegmentation system. These pixels are assigned an intensity value of 0, so that they do not fall within the expected intensity range of the rectum, effectively biasing the autosegmentation algorithm to exclude these pixels from the rectal contour. Figure 3(d) illustrates this approach.

In addition, for the purposes of describing rectal position as a function of slice number, a common landmark is identified from the kVCT prostate contour. The reference MVCT slice, at which the rectal origin is defined for plotting, is the slice containing the most anterior coordinate of the prostate contour.

### 2.4. Contouring algorithm

The basic contouring algorithm used is a 2D version of the Chan-Vese algorithm (Chan and Vese 2001). A key determinant in the effectiveness of the algorithm is the use of a good starting point.

#### 2.4.1. Identification of contour starting point

In an early iteration, the starting point of the rectal contour was identified by scanning for appropriate features, using no *a priori* knowledge, but this was found to be unreliable. The more robust approach adopted here uses the rectal contour manually outlined on the kVCT planning scan as the starting point. Shrinking the original kVCT contour slightly (using erosion with a 3 × 3 structure) allows a ‘bias’ parameter in the autosegmentation algorithm to control the subsequent expansion of the contour.

An improvement to this starting pseudo-contour was implemented by considering shifts of up to 15 mm (20 pixels) in the location of the starting contour, and choosing the starting location with the highest correlation between the shifted contour mask and the MVCT slice being considered. This identified bright regions of the same shape as the kVCT rectal contour within 15 mm (20 pixels) of the starting contour, and accounted for any slice misalignment.

#### 2.4.2. Autosegmentation algorithm

The 2D Chan-Vese algorithm used (Chan and Vese 2001) was implemented as a standard MATLAB function, *activecontour*. Two parameters were critical to the contouring operation: (i) a smoothing parameter, governing the smoothness of the final contour, and (ii) a contraction bias parameter giving the weighting assigned to the area of the contour. Increasingly negative values of contraction parameter encourage expansion of the fitted contour. The values of these parameters were investigated systematically.

### 2.5. Post-processing

Cases were detected where the autosegmentation algorithm did not produce reasonable contours. Post-processing algorithms were therefore developed to identify slices where autosegmentation was poor, and to replace these with an improved estimate of the rectal contour. Autosegmentation contours abutting the edge of the ROI (i.e. the expanded area around the supposed position of the rectum selected for analysis) are rejected as poor contours. Other criteria used to identify erroneous contours are discussed below.

#### 2.5.1. Implausibly large contours and finding the muscle-associated region

**Figure 4.**
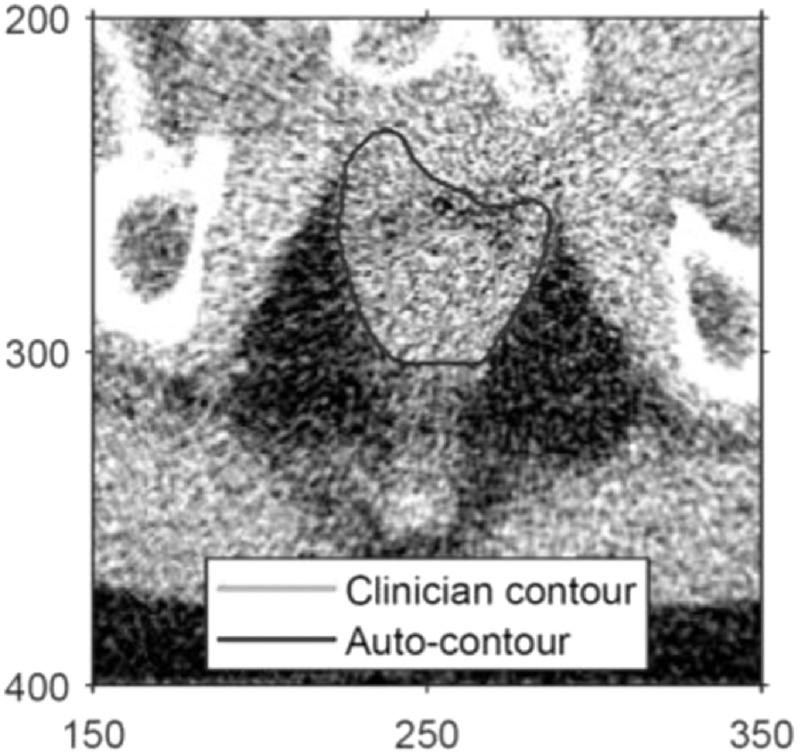
Large autosegmented areas correspond to slices where the rectum is poorly defined, particularly in the lower third of the rectum as illustrated here with an autosegmented area of 1680 mm^2^ (2950 pixels). Note that the shape of the upper edge of the autosegmented contour has been affected by the prostate having been blanked out in the rescaled image (not shown). Axis labels are pixel numbers.

In the lower third of the rectum, the reduced image contrast between the surrounding soft-tissue musculature makes it difficult to distinguish the rectal contour, particularly on lower quality IG scans. In this situation, the Chan-Vese algorithm tends to over-contour. This is illustrated in figure 4, where the autosegmented contour is displayed alongside the clinician-delineated rectal contour. Areas of poor contrast between the rectum and adjacent organs can lead to similarly large and erroneous contours.

**Figure 5.**
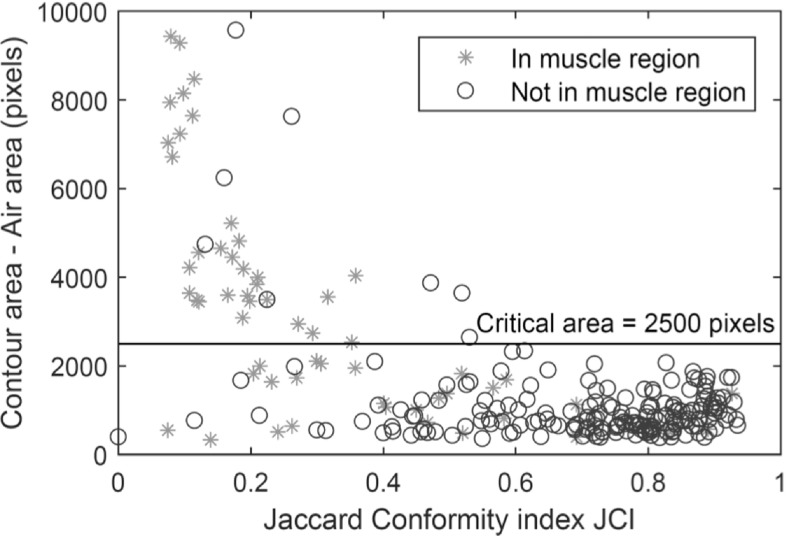
The relationship between the autosegmented rectal contour area (after subtracting the area of air pockets) and the Jaccard Conformity Index, JCI, comparing autosegmented and clinician-outlined rectal contours. Implausibly large areas correlate with scans where the JCI is low. This occurs mainly in the muscle-associated region in the lower third of the rectum, where the algorithm systematically over-estimates the rectal contour (prior to post-processing). The horizontal line shows the cut-off area of 2500 pixels (1420 mm^2^) chosen for implausibly large contour areas.

A threshold value for the contoured area was therefore implemented to identify erroneously large contours. The value of the threshold was identified by considering the relationship between contour area and accuracy of the corresponding autosegmented contour. Accuracy was characterised using the JCI, defined for two contours (in this case comparing autosegmented against clinician contoured) as the intersection area divided by the union area of the two contours. A value of one corresponds to identical contours, and values below 0.5 are relatively poor. Figure 5 shows the relationship between JCI, comparing auto and manual contouring, and the area of the autosegmented region, after subtraction of air. Many of the large contours correspond to the lower third of the rectum where the autosegmentation is systematically over-estimated. A threshold contour area of 1420 mm^2^ (2500 pixels) effectively separates poor quality over-contoured slices from more accurate contours with a higher JCI.

The observation that large errors systematically occur in the muscle region is used to identify the extent of the muscle-associated region in the lower third of the rectum. The top of this muscle-associated region is chosen as the most superior slice with an over-large contour area, but not beyond the 6th slice from the bottom of the MVCT scan. In some cases, slices inferior to this critical slice do not have an over-large contour, but are nevertheless identified as being in the muscle-associated region. In the event of no such slice being found, a default of the second-most inferior slice is chosen as the end of the muscle-associated region.

As a default, contours in this muscle-associated region are taken from the kVCT planning scan, which were manually delineated by the clinician. Two exceptions to this occur when air is present. Where a significant region of air is detected in the MVCT slice, the corresponding air region is used directly as the rectal contour, as previously discussed. Where a significant region of air is detected in a kVCT slice, but there is no air in the corresponding MVCT slice, the original kVCT rectal contour is reduced by an amount corresponding to the air region to produce a best estimate MVCT rectal contour in the absence of air.

#### 2.5.2. Smoothing and interpolation in 3D

Having identified the ‘best estimate’ contours on each slice of the MVCT scan, the three dimensional (3D) structure is assessed to determine whether errors have occurred in the initial autosegmentation. A smoothing interpolation scheme is used to produce a smooth 3D rectal surface from the MVCT contours. This is used to identify, and improve on, erroneous slices. Slices already identified as having a poor contour due to abutting the edge of the search region or with an excessively large contour (whilst outside the muscle-associated lower rectal region) are omitted when calculating this smoothed shape. Contours for each slice are represented in a polar coordinate system where *r* is the distance from the centre and *θ* gives the angular position. Contours are interpolated onto 100 values of *θ*, evenly spaced around the circumference and the origin of each slice is taken as the centroid. Therefore, the full scan can be represented in a *r*-*θ*-*z* coordinate system, where *z* locates the slice position in the cranio-caudal direction. The z-origin is taken as the reference MVCT slice, identified from the kVCT prostate contour as described previously. In this way, the radius *r* is expressed as a function of regularly gridded values of *θ* and *z,* facilitating further analysis. A smooth function is fitted to the radius profile (*r*) in both the circumferential (*θ*) and cranio-caudal (*z*) directions to obtain a new set of values of *r* corresponding to a smoothed shape. These are then converted back to Cartesian coordinates in each slice.

Erroneous slices are identified when the JCI between the smoothed and evaluated autosegmented rectal contours falls below a threshold of 0.5. A second segmentation iteration is then performed on these slices, using the smoothed contour as a starting point. If the second-iteration Chan-Vese contour produces a JCI of greater than 0.5 with the smoothed contour, it is used. If the Chan-Vese contour produces a JCI of less than or equal to 0.5, the interpolated contour taken from the smoothed shape is used instead. If the most superior or most inferior slices are affected, autocontours are replaced by the original kVCT contour, rather than using extrapolation.

**Figure 6.**
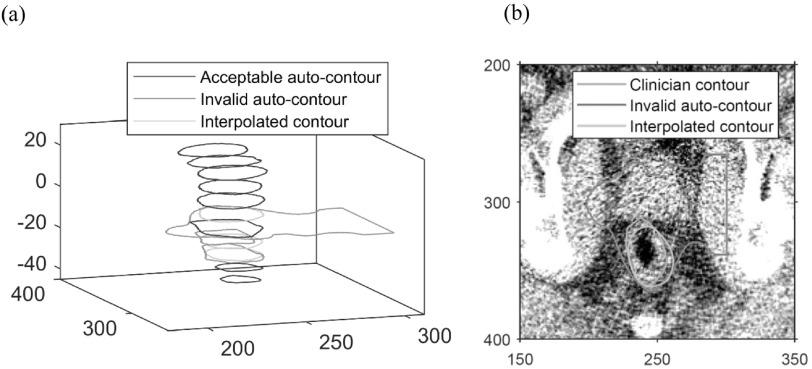
Interpolated contours replace poor-quality autosegmented contours: (a) 3D view, dimensions in millimetres, (b) selected slice, dimensions in pixels.

Figure 6 shows the geometry for a typical case where the interpolation scheme is required. The 3D shape, figure 6(a), illustrates three autosegmented slices that were identified as erroneous. The interpolated contours replace these poor-quality autocontours to give a more anatomically-reasonable overall profile. Figure 6(b) shows these contours and the corresponding clinician contour on an image for one of these slices.

## 3. Results

### 3.1. Training data

**Figure 7.**
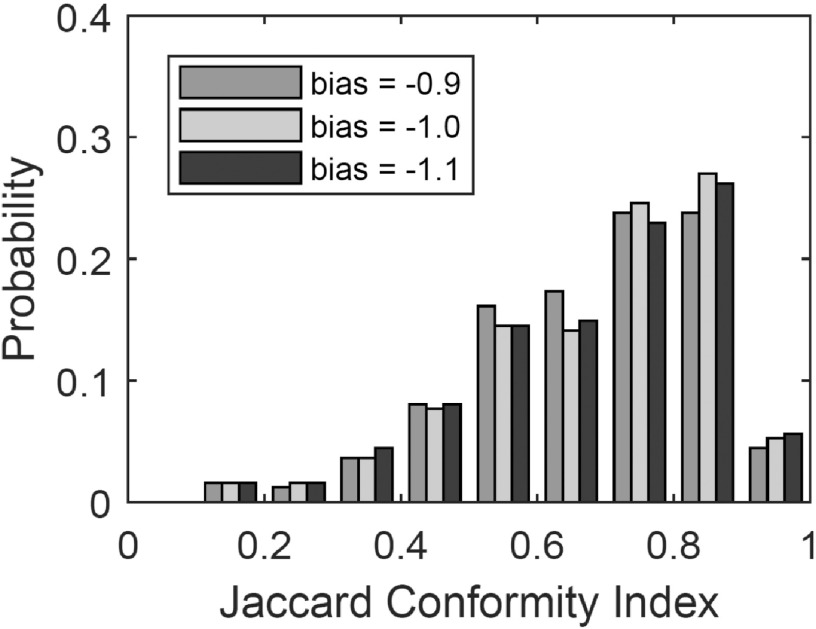
Effect of contraction bias parameter on mean Jaccard Conformity Index, JCI.

A set of 26 training scans was used to develop the algorithm and identify optimal parameter settings. The effect of the contraction bias parameter on the accuracy of the autosegmentation algorithm is shown in figure 7. Mean values for JCI were 0.680, 0.688 and 0.684 for bias values of −0.9, −1.0 and −1.1, respectively. Based on these results, the optimal parameter was taken as −1.0. Similar analyses were used to determine other key values including the smoothing parameter (optimal value found to be 6). Improvements to the algorithm were also implemented based on observations of poor performance in challenging scans. Figure 7 demonstrates the distribution in the accuracy of the contours, with relatively few ‘poor’ contours with JCI below 0.5.

### 3.2. Test results

**Figure 8.**
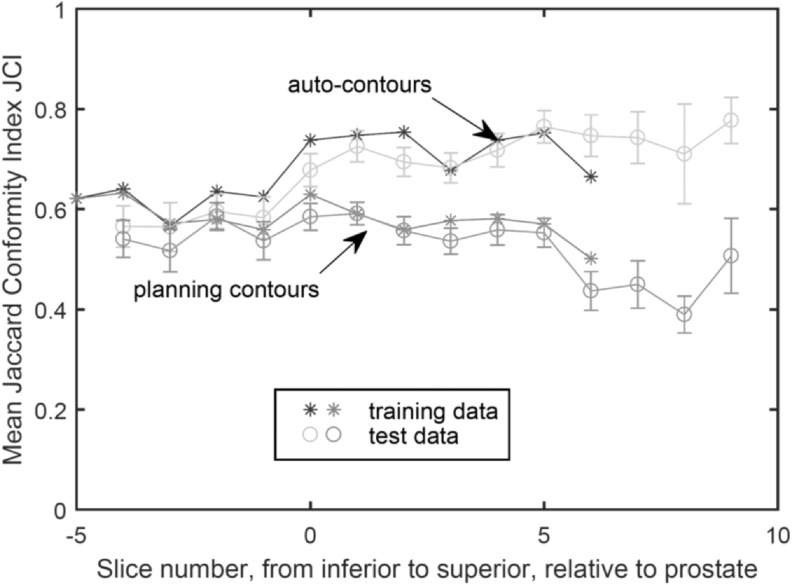
Mean Jaccard Conformity Index, JCI, for training and test data. Clinician-delineated rectal contour on MVCT compared with autosegmented (top two curves) and propagated planning (bottom two curves) rectal contours, as a function of slice location. Slice locations with fewer than five scans (furthest from prostate) have been omitted. Error bars on test data show the standard error.

**Figure 9.**
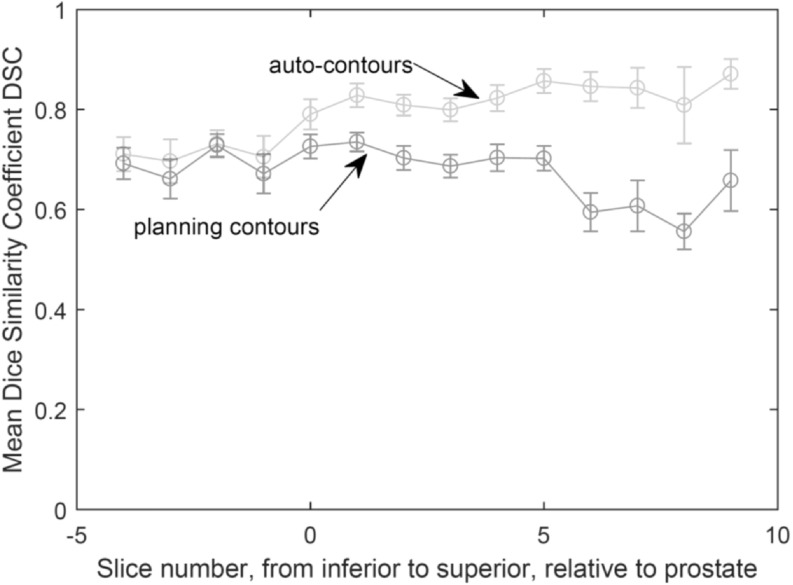
Mean Dice Similarity Coefficient DSC for test data. Clinician-delineated rectal contour on MVCT compared with autosegmented and planning rectal contours, as a function of slice location. Slice locations with fewer than five scans (furthest from prostate) have been omitted. Error bars show the standard error.

**Figure 10.**
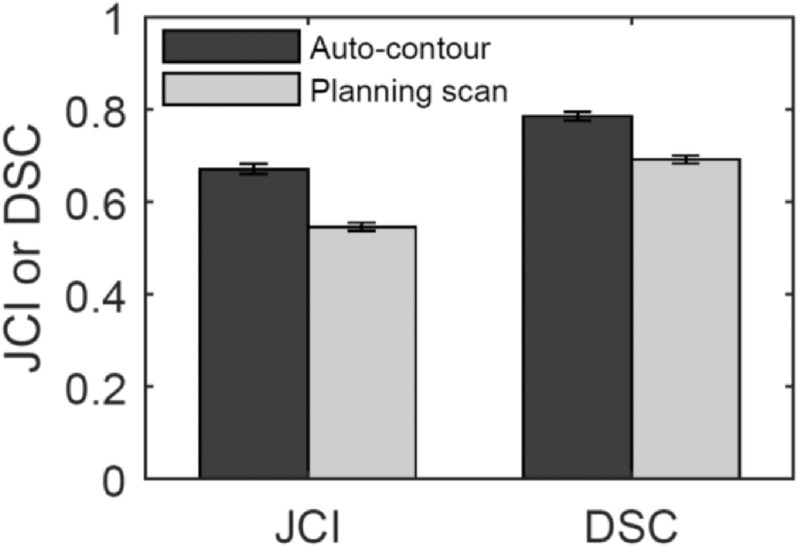
Summary of JCI and DSC results for all slices of test data. Error bars show the standard error (all SE < 0.01).

The algorithm optimised on training data was run on 30 test scans (as discussed in section 2.1). Performance of the algorithm was evaluated by calculating JCI scores for autosegmented contours compared to the gold standard. Dice Similarity Coefficient (DSC) (Dice 1945) scores were also calculated to allow comparison with studies in the literature. JCI and DSC scores for propagated planning contours were also calculated. Figure 8 shows JCI results as a function of slice position relative to the prostate (including training data JCIs for reference, with standard error bars). Slices further from the prostate with fewer than five IG scans were excluded due to being subject to large errors when calculating the mean. The mean JCI scores across all slices from the autosegmentation algorithm were 0.69 and 0.67 for the training and test data, respectively. This is an improvement upon the mean JCI scores from the corresponding propagated planning contours of 0.58 and 0.54 for training and test scans, respectively. The mean DSC for the test data across all slices were 0.78 and 0.69 for the autosegmentation and propagated planning contours, respectively (figure 9). Standard errors are indicated on respective plots. Conformity improves with increasing slice distance from the inferior muscle-associated regions. Training and test data have comparable accuracy. Figure 10 summarises these conformity index results (both JCI and DSC) for autosegmentation of the test data as compared with simple propagation of planning scan contours.

**Figure 11.**
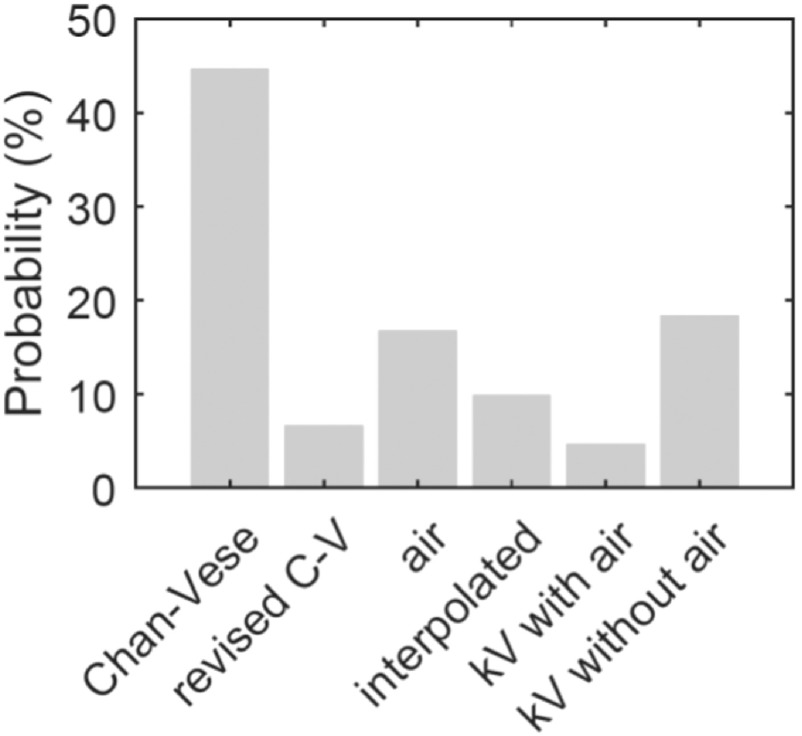
Relative frequencies of strategies deployed in the autosegmentation of the test set.

**Figure 12.**
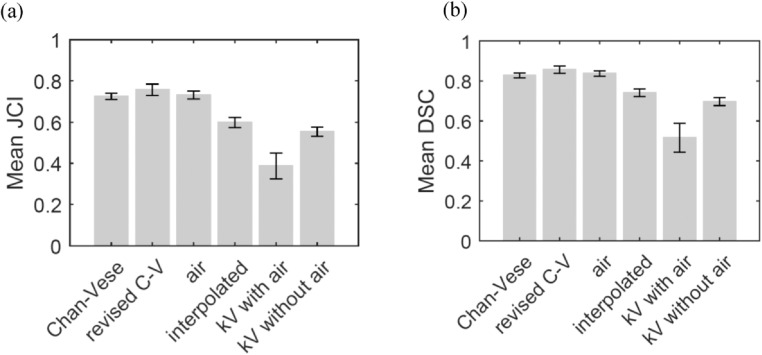
Accuracy of the different autosegmentation methods used in ‘best estimate’ of rectal contour per slice, in the test set: (a) JCI, (b) DSC (error bars indicate standar error).

Figures 11 and 12 give a further breakdown of the underlying processes informing the contours from the test set. Figure 11 shows the probability associated with each method used to estimate the final contour. The large majority of the slices use the Chan-Vese algorithm to estimate the contour. The kV planning scan is chosen as the best estimate for a significant number of cases in muscle-associated regions, where poor contrast dominates. Air-correction also plays a role in determining the final contour in a significant number of cases. The smoothing/interpolation aspect of the algorithm is used less frequently. Although this has a relatively small impact on the accuracy of the results, this process ensures that the resulting 3D shape is smooth and hence anatomically reasonable.

Figure 12 shows the mean accuracy associated with the different autosegmentation methods. Where the Chan-Vese algorithm is used (either on the first or second iteration), the JCI values exceed 0.7. Contours for slices with large air regions perform similarly. By detecting values where slice contours do not fit the smoothed 3D shape, and replacing them with an interpolated contour, an improved estimate of the best contour is achieved. Without this step many of the 3D structures would be much poorer; this step acts as effective ‘disaster mitigation’. The worst cases are those in the poor-contrast muscle-associated region of the rectum, where the autocontouring is not effective and the kV contours are used. In the relatively few cases where there is air in the kV planning scan but not in the daily IG scan, the simplified approach of reducing the area of kV contours by the amount of air does not produce accurate results. A more sophisticated approach, for example using an anatomically-based deformation model, may improve these cases.

## 4. Discussion

An autosegmentation algorithm was developed to identify rectal contours on daily MVCT scans for patients undergoing prostate IGRT. This novel approach involves primary segmentation rather than DIR, as DIR can struggle when dealing with large magnitudes of rectal deformation and varying intensities of rectal contents from day to day. The method uses a modified 2D Chan-Vese algorithm (Chan and Vese 2001), with HU/intensity scaling and additional self-checks. Slices affected by poor contrast, particularly the lower rectal third and surrounding musculature, are detected automatically and replaced by propagating the corresponding kV planning contour as a best estimate. Post-processing identifies erroneous contours and regenerates reasonable estimates via 3D interpolation. The algorithm is a crucial component, integrated within a wider automated processing system, in the calculation of delivered dose to the rectum within the VoxTox research programme (Scaife *et al* 2015, Burnet *et al* 2017, Shelley *et al* 2017).

The autosegmentation algorithm was optimised for identifying the rectal contour on MVCT imaging using a training set of 26 scans from 10 patients. Specific parameters such as HU scaling, identification of air, and selection of image analysis parameters were optimised by trial and error, or based on observation, so may not represent a ‘global minimum’. However, we expect that the algorithm could be adapted for other imaging modalities, and even further anatomical sites.

Validation was performed on 30 test scans. Performance of the autosegmentation test set with respect to the gold standard produced a mean DSC of 0.78 (SE < 0.01). This compared favourably with studies in the literature that used higher quality imaging (discussed previously); kVCT registration (DSC 0.74) (Geraghty *et al* 2013), CT-on-rails DIR/IMM (DSC 0.51/0.71) (Gao *et al* 2006), CBCT modified Demons algorithm (DSC 0.77, Thor *et al* 2011, and DSC range 0.72 to 0.85, Thor *et al* 2013). When comparing these results, it should be noted that Geraghty *et al* used contours from multiple observers, which may result in a pessimistic value of DSC compared with results based on a single observer.

Our experience suggests that in regions of poor image contrast, such as the lower rectal third, the autosegmentation algorithm could be complemented through the use of DIR. Use of a fully 3D algorithm, or a machine learning approach, may improve the accuracy of rectal autosegmentation.

By implementing a 3D interpolation, not only has it been possible to automatically detect erroneous contours, but also the resulting estimated shape is relatively smooth and hence more anatomically representative. Future work will explore using the autosegmented 2D rectal contours as input to a 3D finite element model, allowing biomechanical expansion and voxel-by-voxel tracking, for improved accuracy of delivered dose calculation.

The autosegmentation algorithm has been successfully implemented to accumulate delivered dose, accounting for interfraction motion, based on daily MVCT imaging (Scaife *et al* 2015). It has been a vital tool in testing the hypothesis that delivered dose can be a better predictor of rectal toxicity than planned dose within the VoxTox research programme (Shelley *et al* 2017). This novel approach for autosegmentation of IG scans may contribute to future advances in ART.
